# Usefulness of Tissue Biomarkers versus Prostate-Specific Membrane Antigen-Positron Emission Tomography for Prostate Cancer Biochemical Recurrence after Radical Prostatectomy

**DOI:** 10.3390/cancers16162879

**Published:** 2024-08-19

**Authors:** Gabriela Vera, Pablo A. Rojas, Joseph B. Black, Ignacio F. San Francisco

**Affiliations:** 1 Servicio de Urología, Complejo Asistencial Dr. Sotero del Rio, Santiago 8207257, Chile; gp.veravarela@gmail.com (G.V.); pablorojasruz@gmail.com (P.A.R.); 2 Division of Urologic Surgery, Beth Israel Deaconess Medical Center, Harvard Medical School, Boston, MA 02215-5400, USA; jbblack@bidmc.harvard.edu

**Keywords:** prostate cancer recurrence, novel images, biomarkers

## Abstract

**Simple Summary:**

Patients undergoing definitive prostate cancer therapy experience recurrence despite standard-of-care interventions. Biochemical recurrence is detected initially via prostate-specific antigen (PSA) monitoring and is defined as biochemical recurrence. The clinical importance of the early detection of biochemical recurrence is underscored in this study. This review seeks to further elucidate modalities used for earlier identification of biochemical recurrence. Specifically, we highlight in this review the clinical utility of both tissue biomarkers and prostate-specific membrane antigen-positron emission tomography (PSMA PET) in monitoring patients for recurrence. The summarization provided in this article can help guide clinicians in understanding the recent developments in this field.

**Abstract:**

Despite curative-intent local therapy, approximately 27% to 53% of prostate cancer (PCa) patients experience prostate-specific antigen (PSA) recurrence, known as biochemical recurrence (BCR). BCR significantly raises the risk of PCa-related morbidity and mortality, yet there is no consensus on optimal management. Prostate-specific membrane antigen-positron emission tomography (PSMA PET) has emerged as highly sensitive imaging, distinguishing local recurrences from distant metastases, crucially influencing treatment decisions. Genomic biomarkers such as Decipher, Prolaris, and Oncotype DX contribute to refining recurrence risk profiles, guiding decisions on intensifying adjuvant therapies, like radiotherapy and androgen deprivation therapy (ADT). This review assesses PSMA PET and biomarker utility in post-radical prostatectomy BCR scenarios, highlighting their impact on clinical decision-making. Despite their promising roles, the routine integration of biomarkers is limited by availability and cost, requiring further evidence. PSMA PET remains indispensable for restaging and treatment evaluation in these patients. Integrating biomarkers and PSMA PET promises to optimize personalized management strategies for BCR, though more comprehensive consensus-building studies are needed to define their standardized utility in clinical practice.

## 1. Introduction

Prostate cancer (PCa) is the second most diagnosed cancer worldwide, with an annual incidence of 1,467,854 and 397,430 deaths in 2022 according to the Global Cancer Observatory (GLOBOCAN). In the United States, it represents the most common cancer in men and the second leading cause of cancer death, with over 30,000 deaths annually [1].

One of the curative-intent treatment options for localized PCa is radical prostatectomy (RP). Radical prostatectomy has been evaluated in multiple studies. The SPCG-4 trial demonstrated that RP resulted in a significant reduction in death from any cause at 18 years (RR 0.7, and 95% CI: 0.53–0.95), death from PCa (RR 0.38, and 95% CI: 0.23–0.62), and distant metastasis (RR 0.49, and 95% CI: 0.32–0.74) [2]. In the PIVOT trial, RP significantly reduced mortality from any cause (HR 0.69, and 95% CI: 0.49–0.98), while a meta-analysis based on the findings of these two studies demonstrated the benefit of RP over observation with a significant decrease in death of 9% and disease progression of 43% in PCa patients [3].

Despite undergoing curative-intent local therapy, many men develop recurrence of a prostate-specific antigen (PSA), which is known as biochemical recurrence (BCR). BCR represents the primary form of failure after primary treatment with curative intent. There is a heterogeneity of causes of BCR, ranging from micrometastases, recurrent or regional localized disease, and distant disease (or more than one of these), which increases the risk of prostate cancer-related disease and death [4,5,6,7]. Furthermore, because PSA is not cancer-specific, it can increase due to recurrent benign prostatic tissue that may grow following other curative-intent treatments, such as external beam radiotherapy (EBRT) or minimally invasive therapies, and, on rare occasions, residual benign prostatic tissue post-RP, leading to false positives [8,9].

### 1.1. Why Is It Important to Detect and Manage BCR Appropriately?

BCR occurs in 27 to 53% of PCa patients within 10 years following RP or EBRT [10,11]. In many cases, BCR can occur with only an elevation in PSA, remaining in this state for more than 5 years in a large percentage of patients, without developing clinical or radiological progression and with the patients remaining in good health and asymptomatic for a long time [12]. However, the importance of detecting, staging, and managing these patients appropriately is underscored by studies demonstrating worse oncological outcomes. In a systematic review by Van den Broeck et al., patients with BCR had a higher risk of developing distant metastases, prostate cancer-specific mortality (PCSM), and overall mortality (OS) [13]. In this review, OS in BCR was highly variable with HRs ranging from 1.03 (95% CI 1.004–1.06) to 2.32 (95% CI 1.45–3.71) after primary RP [14,15]. Given this variability in statistical reports, it is difficult to define the true impact of BCR on OS [16]. Additionally, the risk of developing distant metastases in general occurred after 8 years. Recently, Falagario et al. [17] evaluated the association of BCR with PCSM in 16,311 cases of PCa patients. The cumulative incidence at 10 years of PCSM post-RP was 4% (95% CI, 2–6%) in low-risk BCR (Gleason score < 8 and PSADT > 12 mo) and 9% in high-risk BCR (Gleason score ≥ 8 and PSADT ≤ 12 mo).

Moreover, it has been observed that the evolution of patients is different when adding various factors to the disease, and not all of them should undergo identical management, as there is a risk of overtreatment and altering their quality of life. However, the management strategy is still not well defined, so various tools have been sought in recent times to improve these decisions. Among these are the International Society of Urological Pathology (ISUP)’s histological grading system, prostate-specific antigen doubling time (PSADT), conventional imaging, next-generation imaging, and finally genomic testing [18,19,20]. Thus, a PSA doubling time less than 9 months is considered at higher risk of progression and increased risk of death from prostate cancer [21,22]. In fact, men with a PSADT less than 3 months have a median survival of 6 years after BCR [23]. 

As previously mentioned, in the BCR scenario, the utility of imaging has been studied for the early and timely detection of local recurrences and distant disease. In this context, next-generation imaging such as prostate-specific membrane antigen-positron emission tomography (PSMA PET) has been shown to be the most sensitive imaging at low PSA levels (<0.5 ng/mL), helping to distinguish recurrences confined to the prostatic fossa from those that are distant metastases, which can impact therapeutic decision-making in these patients [24]. Additionally, the NCCN [19] and AUA [25] recommend the use of biomarkers, through genomic tests such as Decipher, Prolaris, and Oncotype Dx, to contribute to the overall recurrence risk profile in patients, contributing to the decision to intensify adjuvant therapies. The aim of this review is to evaluate the utility of PSMA PET and biomarkers in the BCR post-RP scenario.

### 1.2. How Is BCR Defined?

BCR is defined in the post-RP context as at least two PSA levels equal or above 0.2 ng/mL, and for post-EBRT, the Phoenix criteria are used, which require a PSA elevation of at least 2 ng/mL above the post-radiation nadir [26].

### 1.3. Why Use Risk Stratification in BCR?

Risk classification was based on the study by Van den Broeck et al. [16], which found that the ISUP histological grade, shorter time from treatment to recurrences, and shorter PSADT were associated with higher PCSM in BCR patients. Therefore, clinical guidelines suggest that patients with BCR should be stratified by progression risk before starting therapies as treatment for recurrence, classifying post-RP BCR patients as high risk if they have either a PSA doubling time (PSADT) ≤ 1 year or Gleason score (GS) of 8–10. Low-risk patients are those with a PSADT > 1 year and GS < 8 [27]. Post-EBRT BCR patients are defined as high risk for disease progression if they have BCR with a PSADT ≤ 18 months and GS 8–10 and low risk if they have a PSADT interval > 18 months and GS < 8 [28]. 

### 1.4. What Are Biomarkers and Which Ones Are Available to Assess BCR?

Biomarkers are molecules that can be used in various PCa scenarios, based on the presence of specific cell types, proteins, metabolites, RNA, DNA mutations, polymorphisms, or epigenetic modifications [29]. Their implementation in PCa has rapidly expanded in the last decade. We previously have described the role of biomarkers in active surveillance [30] and predicting metastatic disease in prostate cancer [31]. While PSA is a widely used tumor marker in this context, it is not cancer-specific, so elevated PSA levels can often be due to other conditions, such as benign prostatic hyperplasia or inflammatory/infectious processes of the prostate. In this context, other molecular markers have been sought for use in PCa, leading to the study of genomic markers.

There are three scenarios in which biomarkers can be used in prostate cancer: in early detection, recent diagnosis, and post-treatment. For these purposes, biomarkers can assess patients who will require biopsy, can select patients for active surveillance or radical therapies, and can be used for adjuvant therapies after curative therapies in patients at high risk of progression.

In this review, we will focus mainly on their utility in evaluating BCR post-RP. The following biomarkers will be addressed in this review:

#### 1.4.1. Prolaris (Myriad Genetics, Salt Lake City, UT, USA)

This is a genomic expression marker that evaluates 31 cell cycle progression (CCP) genes and 15 housekeeping genes, both in pre-biopsy and post-RP specimens. This biomarker was initially developed for breast cancer studies and was later validated for PCa. The test is performed on prostate biopsies for patients with a recent PCa diagnosis (Prolaris biopsy test) and on post-RP specimens (Prolaris post-prostatectomy test). The latter reports the risk of biochemical recurrence at 10 years [32]. The CCP score is an indicator of the proliferative index, which reports a range from 0 to 10. A higher score indicates a more aggressive cancer and a higher risk of disease progression. A one-unit increase is reflected in a doubling of the gene expression level, suggesting a more aggressive tumor [32].

The utility of Prolaris was first reported in a retrospective [33], where there were two cohorts: on one hand, 366 patients underwent RP, and on the other hand, 337 underwent conservative management with transurethral resection of the prostate (TUR-P). The primary endpoint was to evaluate the time to biochemical recurrence for the post-RP cohort and the time to cancer death for the post-TUR-P cohort. In this study, it was found that the CCP score was associated with a higher risk of BCR (HR 1.77, 95% CI 1.40–2.22, and *p* < 0.001) in the post-RP cohort and PCSM in the TUR-P cohort (HR 2.57, 95%CI 1.93–3.43, and *p* < 0.001) [33]. A study by Koch et al. showed that men with higher CCP scores who had BCR post-RP had a higher risk of systemic disease, suggesting that this patient population could benefit from early adjuvant therapy [34].

The Prolaris post-prostatectomy test was subsequently validated in another independent cohort of 413 men in 2013. In this study, it was demonstrated that when clinical–pathological factors are controlled, the CCP score was a strong predictor of BCR (HR 2.1, 95%CI 1.6 to 2.9, and *p* < 0.001). Based on these findings, Prolaris can be used to select patients who are post-RP candidates for adjuvant therapy [35]. 

Several studies have demonstrated an association with BCR and PCSM rather than a recommendation for specific treatments. Brishoff et al. [36] retrospectively applied this score to needle biopsies of men undergoing RP and demonstrated that Prolaris is an independent predictor of biochemical recurrence (HR per score unit 1.47, 95%CI 1.23–1.76, and *p* < 0.001) and metastatic progression (HR per score unit 4.19, 95%CI 2.08–8.45, and *p* < 0.001) [36].

Regarding the use of this biomarker to change treatment behavior in PCa, the PROCEDE-1000 study, a prospective registry with nearly 1600 participants, showed that the CCP score resulted in a treatment change for 47.8% of patients [37]. More specifically, treatment was escalated in 25% of cases and de-escalated in 75%.

Despite this, there are still no prospective data showing clinical superiority when changes in behavior are guided by this specific test [37]. In the latest updates of the clinical guidelines, the EAU [24] mentions that this test still lacks sufficient evidence to be used routinely in the BCR scenario; however, it suggests its use in the decision to perform active surveillance (AS) or to intensify therapies with androgen deprivation therapy (ADT) in PCa. In the case of the NCCN [19], the Panel recommends it in a similar context. According to the above, further randomized prospective studies are required to recommend this biomarker in the BCR scenario.

#### 1.4.2. Oncotype Dx Prostate (Genomic Health, Redwood City, CA, USA)

This is a marker that uses 12 genes related to cancer in four biological pathways (stromal response, androgen signaling, proliferation, and cellular organization) and 5 guardian genes to predict aggressiveness based on pathological findings in radical prostatectomy. The test provides the Genomic Prostate Score (GPS) on a scale of 0 to 100, which corresponds to an increased risk of adverse pathology post-RP.

This test was originally used in the context of breast cancer and was later adapted for PCa, and it was approved for use in this scenario in 2013. Unlike other biomarkers that are only associated with long-term clinical outcomes, the GPS can also predict adverse tumor pathology (extraprostatic extension, positive surgical margins, and seminal vesicle invasion).

In a study by Cullen et al. [38], 431 post-RP biopsies were analyzed to assess the relationship between the GPS score and oncological outcomes. It was found that the GPS was a predictor of BCR (for each 20-unit increase in the GPS, HR 2.73, 95% CI 1.84–3.96, and *p* < 0.001) as well as a significant predictor of metastasis-free survival (HR/20 units 3.83, 95% CI 1.13–12.60, and *p* = 0.032) in univariate analysis. Furthermore, the GPS was found to be associated with adverse pathology (primary Gleason 4 or any pattern 5 and/or pT3) after adjusting for NCCN risk (OR/20 3.23, 95% CI 2.14–4.97, and *p* < 0.001). 

Covas et al. [39] evaluated 749 patients who underwent the Oncotype DX assay followed by radical prostatectomy (RP), demonstrating a significant association between the GPS and adverse pathology post-RP. In a multivariate analysis, for every 20-point change in the GPS, the GPS was an independent factor for extraprostatic extension (odds ratio (OR) 1.8 and 95% CI, 1.4–2.3) and seminal vesicle invasion (OR 2.1, and 95% CI 1.3–3.4).

Regarding clinical decision change based on the use of Oncotype DX, Badani et al. conducted a prospective study of 158 men with very low to low–intermediate risk, resulting in a change in clinical management in 18% of cases. Specifically, AS increased from 41 to 51%, RP decreased from 21 to 19%, and EBRT decreased by 33% [40].

The NCCN [19] and EAU [24] guidelines recommend this biomarker in specific situations as mentioned earlier for Prolaris. However, the American Society of Clinical Oncology (ASCO) [41] guidelines discourage its routine use, recommending it only when its results, combined with other clinical factors, may lead to a change in clinical behavior. Similarly to Prolaris, its use in BCR is not recommended by the ASCO, and further prospective studies are needed to demonstrate the consequences of its use regarding oncological outcomes.

#### 1.4.3. Decipher (Genome Dx Biosciences Based in Vancouver, BC, Canada, and Mayo Clinic)

This is a 22-gene genomic classifier initially developed for the post-RP setting. It applies a whole-transcriptome microarray analysis using a random forest algorithm based on the expression of 22 RNA biomarkers related to androgen receptor signaling, cell proliferation, differentiation, motility, and immune modulation.

The Decipher score ranges from 0 to 1, with higher values indicating more aggressive disease, with cutoff points of 0.45 and 0.60 to categorize patients as low, intermediate, and high risk. Decipher was approved in the United States to assess the risk of BCR or clinical progression (metastasis) in post-RP patients with adverse pathology (pT3 and/or positive margins or biochemical failure) or for PSA recurrence during follow-up [42,43]. 

Decipher has been applied in scenarios where management change is possible, including low-risk disease (conservative therapy vs. radical treatment), intermediate disease (to guide the addition of androgen deprivation therapy to radiotherapy), the post-RP scenario (to guide adjuvant radiotherapy), post-RP recurrence (to guide the addition of androgen deprivation therapy with salvage radiotherapy), and more advanced scenarios (hormone-sensitive metastatic and non-metastatic hormone-resistant scen), where low genomic classifier (GC) values predict a more favorable prognosis and may change the addition of intensifying treatment [44].

In a systematic review, it was demonstrated that in patients with high-risk disease in GC with BCR, the biomarker proved to be an independently associated factor in developing metastatic disease and PCSM [44]. A prospective study revealed that within patients with high GC risk, those who underwent adjuvant radiotherapy had a lower PSA recurrence at 2 years compared to those who did not receive adjuvant radiotherapy (3% vs. 25%, *p* = 0.013). However, there were no differences at 2 years between those who underwent observation versus those who received adjuvant radiotherapy within patients with low–intermediate GC risk (3% versus 0.5%, *p* = 0.77) [45].

The studies conducted by Berlin et al. [46] and the post hoc analyses of the phase 3 randomized RTOG 9601 study [47] confirmed that patients could safely avoid intensified treatment with ADT in the BCR scenario: both studies demonstrated that the Decipher biomarker was associated with distant metastasis or cancer-specific mortality. In the RTOG 9601 study, 486 BCR patients were evaluated [48]. A multivariate analysis showed that the GC score (as a continuous variable per 0.1 unit) was independently associated with a higher risk of distant metastasis (HR 1.17, 95% CI 1.05–1.32, and *p* = 0.006), PCSM (HR 1.39, 95% CI 1.20–1.63, and *p* < 0.001), and OS (HR 1.17, 95% CI 1.06–1.29, and *p* = 0.002). In this same study, it was found that patients with a low GC score apparently would not benefit from intensified treatments. In this context, the results demonstrate that the GC score could identify BCR patients, regardless of PSA level, who would or would not benefit from salvage EBRT (sEBRT) + ADT versus sEBRT alone.

In a meta-analysis by Spratt et al., the 10-year cumulative incidence of metastasis was evaluated according to the GC risk categories post-RP, resulting in 5.5%, 15.0%, and 26.7% for low, intermediate, and high risk, respectively (*p* < 0.001) [49]. Dal Prana et al. evaluated samples from 226 post-RP patients to determine the association of GC with 5-year freedom from biochemical progression [50]. Patients with high-risk GC had a 5-year freedom from biochemical survivor rate of 45% (95% CI 32–59%) versus 71% (95% CI 64–78%) in the low–intermediate GC group.

Regarding the use of Decipher to change management in BCR patients, the PRO-ACT study prospectively evaluated the decision changes of 15 community urologists before and after using Decipher [51]. It was observed that there was a management change to adjuvant EBRT (aEBRT) in 30% of cases, and 42% of patients who were initially recommended adjuvant therapy were reassigned to observation. The decision change in adjuvant therapy use with Decipher in this study was statistically significant (*p* < 0.001). Another study that evaluated this aspect was PRO-IMPACT, which showed similar results [52]. In this prospective study of 265 post-RP patients, adjuvant therapy use was evaluated. After the use of Decipher testing, 18% of treatment recommendations changed to aEBRT and 32% to salvage therapy. In both groups, Decipher testing was associated with a significant decrease in decisional conflicts for both patients and physicians (*p* < 0.001). Marascio et al. [45] also prospectively evaluated outcomes in BCR patients who received post-RP EBRT based on adverse pathological presentations, finding that the GC changed management in 39% of patients. Those with high genomic risk were recommended for aEBRT, and in these patients, the 2-year BCR rates in those who followed the oncology committee’s recommendations were 3% compared to 25% in those who did not (*p* = 0.01). On the other hand, those with low–intermediate genomic risk were all recommended for observation with sEBRT as needed. In these patients, the 2-year BCR rates were 3% and 0.5% for those who underwent aEBRT (*p* = 0.77).

There are no prospective studies that have evaluated the effect of management changes on patient outcomes. However, Den et al. [53] demonstrated in a prospective study that among patients with intermediate-to-high GC who underwent early sEBRT (PSA ≤ 0.2 ng/mL) versus those who did not, the 8-year cumulative metastatic disease rates were 3% versus 23%. These rates were not different in low GC patients with early sEBRT use. Additionally, Feng et al. [48], using data from the RTOG 9601 study, showed that patients with low GC scores had minimal benefit in adding hormonal therapy alongside early sEBRT, with a 0.4% reduction in metastasis at 12 years. Meanwhile, for patients with intermediate or high GC scores, the effect was substantial, with an 11.2% reduction in the 12-year metastasis rate by adding hormonal therapy to sEBRT.

In clinical guidelines, in addition to the recommendations mentioned earlier where other biomarkers previously addressed in this review could be used, in the case of Decipher, there are some additional recommendations. The NCCN [19] indicates that it can be used in patients with adverse post-RP presentations to evaluate adjuvant treatment and may be considered as part of counseling for risk stratification in patients with BCR post-RP. Both the AUA [25] and EAU [24] allude to the need for further studies to recommend it but see that in the future it may contribute to decision-making regarding ADT intensification to salvage radiotherapy, or to the decision to initiate EBRT in the context of BCR.

In the future, genetic markers like Decipher could be routinely used to guide adjuvant use in high-risk BCR patients, as although there is currently no consensus on this, it seems that this could help reduce the risk of overtreatment and avoid delaying therapies that could benefit patients in this scenario. However, the expense and availability of biomarkers present a significant challenge that needs to be addressed. Currently, the biomarkers available on the market are priced at around USD 2000 [31], which poses a substantial barrier, particularly in developing countries.

Table 1 summarizes the ability to predict BCR of the different biomarkers presented previously and compares it with PSMA PET. In addition, it shows the ability to change treatment.

## 2. PSMA PET

### 2.1. Use of PSMA PET in PCa, Utility and Impact on Clinical Practice

Traditionally, in PCa, conventional imaging modalities such as Computed Tomography (CT) and bone scan have been used for staging and assessing metastasis or recurrence. However, there has been increasing interest in the use of other imaging studies, such as PET. Specifically in PCa, PSMA, a transmembrane protein present in prostatic tissue with increased expression in PCa, has been studied. Its use has primarily increased to determine the presence of local recurrence, nodal involvement, and distant metastasis, which are key questions in BCR to define the course of action. Currently, five PET tracers have been approved by the Food and Drug Administration (FDA) for use in PCa patients: Ga-68gozeteotide (Ga-68 PSMA-11), F-18 piflufolastat (DCFPyL), C-11 choline, F-18 fluciclovine, and F-18 sodium fluoride. All these radiotracers have been approved for use in evaluating BCR, while Ga68 PSMA-11 and F-18 piflufolastat are also approved for patients at initial staging with suspected metastatic disease.

In the context of BCR, the radiotracers DCFPyL and Ga-68 -PSMA-11 have been evaluated in phase III studies in the BCR setting, with positive predictive values ranging between 84% and 92% [54,55]. Seifert et al. achieved similar sensitivity with the use of the radiotracer F-18 fluciclovine, also demonstrating better detection of bone metastases than the bone scan, mainly because it detects early bone infiltration before the occurrence of osteoblastic reactions [56]. In a recent systematic review [57] including 20 prospective studies, the role of Ga-68 and F-18 PSMA PET/CT in 2110 BCR patients was evaluated, with PSMA PET positivity at 66.6%, significantly depending on the PSA levels at the time of analysis. Post-RP, Ga-68 PSMA-11 PET demonstrated good detection rates even with low PSA levels. This was demonstrated by De Visschere et al. with positivity rates ranging from 11.0% to 65.0% with PSA values < 0.5 ng/mL [58]. The largest series published by Afshar-Oromieh et al. reported 43.3% detection in 226 patients with PSA ≤ 0.2 ng/mL post-RP [59].

In studies evaluating lesion detection in BCR, PSMA PET showed improved lesion localization after radical therapy [60]. In comparative studies of conventional imaging versus PSMA PET, the latter improved detection rates by an absolute magnitude of 34–59% [61,62,63]. However, PET/CT scans using Ga-68 or F-18 tracers often fail to detect suspicious lesions in a significant number of cases of BCR, particularly when PSA levels are low. For instance, Afshar-Oromieh and colleagues reported a 42.5% rate of negative scans in individuals with PSA levels between 0.2 and 0.5 ng/mL and a 27.8% rate in those with PSA levels > 0.5 to ≤1.0 ng/mL [59]. Recent studies have evaluated the use of PSMA PET in BCR. EMPIRE-1, a single-center, open-label, phase II/III study, randomized 365 BCR post-RP patients into two staging groups: those with negative conventional images and those with negative conventional images plus F-18 fluciclovine-PET. After a median follow-up of 3.5 years, the PET group had significantly greater event-free survival (HR: 2.04, 95% CI: 1.06–3.93, and *p* = 0.0327) [64]. The ORIOLE trial showed that patients with positive disease in PSMA PET who were not included in the treatment plan experienced faster disease recurrence than those with covered disease. These studies aimed to evaluate the use of PSMA PET for managing lesions detected in imaging in patients with BCR [65].

### 2.2. Use of PSMA PET as Metastasis-Directed Therapy (MDT)

MDT involves the use of therapy directed at metastases, either with surgery or stereotactic body radiation therapy (SBRT), in the setting of oligometastatic patients. The definition of oligometastasis for MDT is not well established, but it is generally accepted that it involves five or fewer lesions. Studies on this treatment modality have emerged hand in hand with the use of PSMA PET, as it would improve lesion detection in these patients, initially with the primary goal of delaying the start of ADT with eventual improvement in oncological outcomes. Most trials investigating MDT have predominantly utilized stereotactic body radiotherapy (SBRT) rather than surgery [65,66].

The main study supporting MDT is the STOMP trial, which randomized BCR patients into observation versus MDT therapy, based on choline PET imaging. The primary outcome of the study was to evaluate ADT-free survival. A median ADT-free survival of 21 months was reported for the MDT group and 13 months for the observation group (HR 0.6; 95%CI, 0.4–0.9) [66]. Building off the STOMP trial, the EXTEND trial investigated the utility of providing patients with oligometastatic disease with a combination of radiation therapy and intermittent hormonal therapy [67]. Patients were randomized to MDT with intermittent hormone therapy and intermittent hormone therapy alone. EXTEND found that a combination of MDT and intermittent hormonal therapy was associated with an improvement in progression-free survival when compared to hormone-only therapy (*p* < 0.001).

The STOMP trial, along with the ORIOLE and EMPIRE-1 studies, provided the eagerly awaited data demonstrating direct benefit in survival using MDT with PSMA PET findings. The ORIOLE trial [65] found that progression in patients with lesions treated by PSMA PET was 5% versus 38% in men with untreated lesions (*p* = 0.03). Moreover, de novo metastatic lesions that occurred within 6 months represented 15.8% and 62.5% in these groups, respectively (*p* = 0.006). The STOMP trial [66] reinforced these findings, using 11C/18F choline as PSMA PET radiotracers and randomizing patients into an observation group and an MDT group, finding a median ADT-free survival of 13 months for the observation group and 21 months for the MDT group (HR 0.60 with long rank *p* = 0.11). This confirms the benefit of PET-guided treatments in optimizing survival outcomes in BCR patients and ADT-free survival.

### 2.3. Use of PSMA PET in Changing Therapeutic Decisions in BCR Patients

The use of PSMA PET allows for earlier detection in imaging, potentially leading to patient restaging and ultimately changing treatment plans. Patients who would historically be considered N0 and M0 are reclassified as N1 and M1 positive. The CONDOR study, whose primary endpoint was to determine the diagnostic performance of PSMA PET, detected treatment changes based on 64%. It is important to note that not all treatment changes involved escalation to systemic therapy. Specifically, 28% of changes involved the addition of systemic therapy to salvage therapy, but also 21% transitioned from systemic therapy to salvage therapy, 24% involved the initiation of therapy when observation had been planned, and 4% transitioned to observation from planned treatment [54]. Fender et al. reported similar results, with a management change occurring when using PSMA PET in 260 of 382 patients (68%) [68].

### 2.4. What Do Clinical Guidelines Recommend Regarding the Use of PSMA PET in BCR?

The EAU [24] recommends using PSMA PET in BCR with a weak grade. The recommendation is based on PSMA PET being the imaging modality with the highest sensitivity to low PSA levels (<0.5 ng/mL), as well as being able to help distinguish patients with local recurrence from those with distant disease, impacting the use of adjuvant therapies. The NCCN [19] also recommends it as a study in BCR, while the AUA [25] recommends its use with moderate strength for this same scenario.

Table 2 shows the recommended clinical guidelines on biomarkers and PSMA PET in BCR patients.

## 3. Treatment Recommendations for Patients with BCR after Definitive Treatment with RP

The treatment of patients with BCR in PCa is still controversial, given the heterogeneity of the studies involved and their variability. Below are the available therapies.

### 3.1. Salvage Radiotherapy (sEBRT)

It has a potentially curative purpose and delays the onset of ADT. Adjuvant radiotherapy (aEBRT) and sEBRT have shown comparable oncologic outcomes in prospective studies; however, the disadvantage of aEBRT has been associated with greater adverse effects, especially in terms of late genitourinary toxicity and erectile dysfunction [69,70,71]. Boorjian et al. [72] reported a 75% reduction in systemic progression with sEBRT compared to no use of sEBRT. It has also been shown in systematic reviews and meta-analyses that in BCR post-RP, sEBRT has benefits in OS and PCSM [73]. sBERT has been shown to be effective mainly in patients with a shorter PSADT, and those who would mainly benefit are those with rapid PSA kinetics post-RP and a PSA limit of 0.4 ng/mL [16]. The EAU [24] recommendation is sEBRT in BCR with high risk and PSA levels < 0.5 ng/mL, while the AUA [25] recommends it with PSA levels < 1 ng/mL. The NCCN [19] recommends that patients with a high-risk Decipher score should be considered for EBRT with ADT when the opportunity for early EBRT has been lost.

### 3.2. ADT + sEBRT

The logic of adding ADT to sEBRT in these patients would be that it downregulates the vascular endothelial growth factor, leading to apoptosis of endothelial cells and decreased vascularization and reducing the required dose of EBRT [74]. The RTOG 9601 study showed that there would be a benefit of adding bicalutamide for 2 years (150 mg o.d.) to sEBRT [75]. Post hoc studies observed that this benefit occurred only in patients with a PSA > 0.6 ng/mL pre-sEBRT [76]. Something similar was demonstrated in the GETUG-AFU 16 study, a prospective, phase 3 study, in which treatment for 6 months with goserelin with sEBRT significantly improved PFS (*p* < 0.001) and metastasis-free survival (MFS; *p* = 0.034) compared to sEBRT alone after 10 years of follow-up [77]. Although these studies support adding ADT to sEBRT, it should be noted that the radiation dose used in the RTOG 9601 study was suboptimal, as was the technique. For these reasons, it is still not clear which specific patients would benefit from its use, nor which is the most appropriate ADT in this scenario. The EAU [24] suggests that patients at high risk of progression may benefit from two years of ADT, while six months of ADT use may be indicated for those at low risk. Patients at low risk could even receive sEBRT alone. In a subanalysis of men with low PSA (<0.6 ng/mL), there were no improvements in OS (HR: 1.16, and 95% CI: 0.79–1.70), with increased other-cause mortality (sub-distribution HR: 1.94, 95% CI: 1.17–3.20, and *p* = 0.01) and increased odds of late grades 3–5 cardiac and neurologic toxic side effects (OR: 3.57, 95% CI: 1.09–15.97, and *p* = 0.05) [24]. In this scenario, the use of Decipher could help elucidate which patients would benefit from adding ADT.

### 3.3. Systemic Treatment with First- and Second-Generation ADT (with or without EBRT)

The use of ADT alone for BCR was evaluated in a systematic review in patients at high risk of progression [78]. The problem related to the use of ADT is the alteration in quality of life due to associated adverse effects, including depression, fatigue, hot flashes, and sexual dysfunction [79]. In addition, there is a higher risk of cardiovascular disease, diabetes mellitus, and osteoporosis [80,81,82]. The TOAD study evaluated OS with the use of immediate vs. delayed ADT in 293 men [83]. Survival at 5 years was slightly higher in immediate therapy vs. delayed therapy (13% vs. 20%) with a significantly longer time to local progression (adjusted HR 0.51; 95%CI 0.34–0.76; and *p* = 0.001) in immediate therapy. However, the time to distant progression was not statistically significant between both treatments after 6 years of follow-up. Although immediate therapy had a smaller deterioration in quality of life, more patients experienced serious adverse effects compared to delayed therapy (41% vs. 32%). Because of this, because the clinical benefit in OS is modest in immediate ADT and the adverse effects are considerable, it should be considered in patients at high risk.

An alternative to ADT is its intermittent use. Multiple reviews have been published since the classic work of Hussain et al., which found no significant differences in overall survival between patients on continuous ADT compared with intermittent ADT [84]. Patients managed with intermittent ADT show an improvement in mental health and recovery of sexual function, with better erectile function [85]. In addition, patients receiving intermittent ADT had a reduced risk of heart failure and fracture [86]. Another benefit seen with intermittent use is the low risk of developing castration-resistant PCa compared to continuous ADT [87]. In summary, there is a large number of publications confirming the benefits of intermittent ADT in BCR [67,88,89,90]; therefore, it is an alternative in this clinical scenario [91].

Second-generation antiandrogen therapies: As recently published by the EMBARK study [92], the use of enzalutamide in patients with high-risk BCR and negative conventional imaging, who received this drug in conjunction with leuprolide, had significant benefits in metastasis-free survival (MFS) (HR 0.42, and 95%CI 0.30–0.61; *p* < 0.0001) or enzalutamide monotherapy (HR 0.63, and 95%CI 0.46–0.87; *p* = 0.005) vs. placebo combined with leuprolide after 60 months of follow-up.

### 3.4. Monitoring

Monitoring may be an appropriate measure in patients at low risk of progression in BCR, as this would avoid overtreating patients and subjecting them to potentially harmful therapies [19,24,25].

## 4. Conclusions and Future Directions

BCR is common and represents one of the early findings in the failure of curative treatment, occurring in up to half of cases in patients post-RP. Its peculiarity lies in the fact that patients are generally asymptomatic at diagnosis and BCR often only presents as a finding in laboratory tests during PCa follow-up.

Despite this, the evidence previously presented shows that there is a subgroup of patients who benefit mostly from adjuvant therapies in PCa with BCR, while others, who have lower risk, may be subjected to unnecessary treatment. It is for these reasons that BCR must be correctly addressed by clinicians. First, an appropriate diagnosis must be reached, then proper stratification according to the risk of progression must be performed, and based on these findings, therapeutic options must be evaluated, avoiding overtreating patients to thus avoid alterations in quality of life and damage to their health.

The main challenge is to correctly identify patients who will benefit from adjuvant treatment post-RP. On the one hand, there are prognostic histological factors, such as ISUP grade, tumor extension, margin status, and associated nodal involvement. However, these variables, which can be used to make the decision to initiate adjuvant therapies, also carry the risk of overtreatment of patients. There is a wide range of men with BCR who do not experience better oncological outcomes with adjuvant therapies, indicating that more precise tools are still needed to identify them correctly. In this context, the use of biomarkers such as Decipher, Oncotype, and Prolaris has shown their utility as predictors of progression to guide the use of appropriate treatments in BCR. Of these biomarkers, Decipher is the only one recommended in a clinical guideline (NCCN) for use in BCR, potentially guiding the addition of ADT to sEBRT. Regarding Prolaris and Oncotype, their non-routine use could be more suitable in the context of localized disease to define candidates for active surveillance; however, their use is not recommended in BCR due to the lack of available evidence. Additionally, it should be noted that these tests are not widely accessible and have a high cost in the market of approximately USD 2000.

The use of imaging in BCR is essential to evaluate recurrent or distant disease and to assess the treatment to follow, either with EBRT alone or combined with ADT or, as has been developed recently in some studies, MDT. PSMA PET has been shown to improve lesion localization after radical therapy when compared to conventional imaging, with an absolute magnitude of 34–59% [61,62,63]. Despite this, in many cases of BCR, no suspicious lesion is found in the image. Additionally, there are many reasons for uptake that do not represent cancer (false positive), such as some costal lesions with low uptake that should not be considered malignant, as well as hemangiomas, Paget’s disease, and fibrous dysplasia. As such, their interpretation requires trained and experienced radiologists [93,94]. It is important to consider that this examination is not widely available in all centers, and so far, there is no clear consensus on management using this study, especially if it is negative for lesions in the context of BCR. However, given its higher sensitivity, it is the imaging modality of choice in this scenario, supported by clinical guidelines.

Despite the heterogeneity of the evidence, there has been significant progress in the personalized treatment of BCR in the last decade. Due to the lack of consensus among clinical guidelines regarding the most appropriate management, and because the level of type 1 evidence on the treatment for biochemical recurrence is limited, patients with BCR should be managed with tailored therapies according to each case, incorporating different strategies to achieve this goal. The use of biomarkers could have a niche to properly distinguish patients who would benefit from adjuvant therapies or intensification with hormonal therapy in BCR, added to other factors previously mentioned as a set. However, with limitations in availability and cost, more evidence is still needed to recommend their routine use. 

On the other hand, the use of PSMA PET is essential for restaging these patients and evaluating the use of other therapies according to the findings. The use of both biomarkers and PSMA PET is not mutually exclusive for BCR management and, rather, we believe they would have a synergistic effect in the proper decision-making in this context; however, more studies are needed to reach a consensus on their use and utility in this scenario.

## Figures and Tables

**Table 1:** A summary of the BCR prediction capacity, treatment switching capacity, and clinical guideline recommendation of the different biomarkers described and compared with PSMA PET.

	Predictor of BCR	Treatment Change (Pre- or Post-RP)
**Prolaris (Myriad Genetics, Salt Lake City, UT, USA)**	Yes [33,35]	47.8% [37]
**Oncotype Dx Prostate (Genomic Health, Redwood City, CA, USA)**	Yes [38]	18% [40]
**Decipher (Genome Dx Biosciences based in Vancouver, BC, Canada, and Mayo Clinic)**	Yes [48,50]	39% [45]
**PSMA PET**	Yes [54]	64% [54]

BCR: biochemical recurrence. RP: radical prostatectomy. PSMA PET: prostate-specific membrane antigen-positron emission tomography.

**Table 2:** A summary of the clinical guideline recommendation of the different biomarkers described and compared with PSMA PET.

	Recommended by Guidelines to BCR
**Prolaris (Myriad Genetics, Salt Lake City, UT, USA)**	Not routinely [19,24]
**Oncotype Dx Prostate (Genomic Health, Redwood City, CA, USA)**	Not routinely [19,24,42]
**Decipher (Genome Dx Biosciences based in Vancouver, BC, Canada, and Mayo Clinic)**	Yes [19]/not routinely [24,25]
**PSMA PET**	Yes [19,24,25]

BCR: biochemical recurrence. PSMA PET: prostate-specific membrane antigen-positron emission tomography.

## Data Availability

No new data were created or analyzed in this study. Data sharing is not applicable to this article.
